# Health impact of natural gas emission at Cava dei Selci residential zone (metropolitan city of Rome, Italy)

**DOI:** 10.1007/s10653-022-01244-6

**Published:** 2022-03-12

**Authors:** Maria Luisa Carapezza, Luca Tarchini, Carla Ancona, Francesco Forastiere, Massimo Ranaldi, Tullio Ricci, Gabriele De Simone, Francesca Mataloni, Nicola Mauro Pagliuca, Franco Barberi

**Affiliations:** 1 INGV – Istituto Nazionale di Geofisica e Vulcanologia, Sezione Roma 1, Rome, Italy; 2 Department of Epidemiology, Lazio Regional Health Service, Rome, Italy

**Keywords:** Cava dei Selci (Rome), Gas hazard assessment, Soil CO_2_ flux surveys, Indoor concentration of CO_2_ and H_2_S, Gas-related health problems, Epidemiologic study on mortality and ERV

## Abstract

Natural gas hazard was assessed at Cava dei Selci, a residential neighbourhood of Marino (Rome) by a joint study of gas emissions and related health problems. Here a densely urbanized zone with 4000 residents surrounds a dangerous natural gas discharge where, along the years, dozens of animals were killed by the gas. Gas originates from Colli Albani volcano and consists mostly of CO_2_ with ~ 1 vol% of H_2_S. In recent years, several gas-related accidents occurred in the urbanized zone (gas blowouts and road collapses). Some houses were evacuated because of hazardous indoor air gas concentration. Gas hazard was assessed by soil CO_2_ flux and concentration surveys and indoor and outdoor air CO_2_ and H_2_S concentration measurements. Open fields and house gardens release a high quantity of CO_2_ (32.23 tonnes * day^−1^). Inside most houses, CO_2_ air concentration exceeds 0.1 vol%, the acceptable long-term exposure range. In several houses both CO_2_ and H_2_S exceed the IDLH level (Immediately Dangerous to Life and Health). An epidemiological cohort study was carried out on the residents of two Cava dei Selci zones with high (zone A) and medium (zone B) gas hazard exposure, using the rest of Marino as reference zone. We found excess mortality and emergency room visits (ERV) related to high exposure to CO_2_ and H_2_S; in particular, an increased risk of mortality and ERV for diseases of central nervous system (HR 1.57, 95% CI 0.76–3.25 and HR 5.82, 95% CI 1.27–26.56, respectively) was found among men living in zone A.

**Supplementary Information:**

The online version contains supplementary material available at 10.1007/s10653-022-01244-6.

## Introduction

The health effects of gas emissions have been studied mostly for anthropogenic industrial sources (e.g. Martin-Olmedo et al., 2019; Bauleo et al., 2019; Leogrande et al., 2019). Comparatively less attention has been devoted to health effects of natural gases, although carbon dioxide (CO_2_) and hydrogen sulphide (H_2_S) are dangerous gas commonly released in volcanic, geothermal and tectonically active areas of the world (Hansell & Oppenheimer, 2004; IVHHN, 2020). In this paper, we address the health effects of a long-term exposure to these gases in a densely populated zone near Rome, located on the slope of Colli Albani volcano.

Central Italy is a tectonically active volcanic and geothermal region, where an appreciable quantity of deep-originated gas is released to the atmosphere, either convectively through tectonic fractures or diffusively from the soil. The emitted gas is dominated by CO_2_ with a concentration usually over 90 vol%, but it contains an appreciable quantity of H_2_S up to ~ 6 vol% (Carapezza et al., 2019). Being denser than air, both gases tend to accumulate near the ground, particularly in morphological depressions or in house basements, where air concentrations dangerous for human health can be reached.

Hydrogen sulphide (H_2_S) is a toxic gas, with an IDLH level (Immediately Dangerous to Life and Health, i.e. the concentration level that interferes with the ability to escape) of only 100 ppm (OSHA, 2019) and a potentially lethal threshold of 250 ppm (IVHHN, 2020). Carbon dioxide (CO_2_) is an asphyxiant gas, toxic at high concentrations (Knoppel & Schlitt, 1995). A high CO_2_ air concentration implies a correspondent reduction in the oxygen air content. At sea level and dry air, the IDLH level for oxygen is 12.5 vol% (Mc Manus, 2009) and this level is reached with a CO_2_ air concentration of 8.3 vol%. It is worth noting that a CO_2_ air concentration of 8 vol% is considered as the potentially lethal threshold (IVHHN, 2020). An immediate evacuation of indoor spaces is recommended if CO_2_ concentration exceeds 1.5 vol% (the occupational short-term exposure limit value).

The guidelines values and limits for CO_2_ and H_2_S concentrations inside residences indicated by WHO and adopted by the various countries, have been reviewed by ISS (the Italian Health Institute; Settimo et al., 2016). The highest CO_2_ ALTER (Acceptable Long Term Exposure Range) for residential indoor air is 3500 ppm (0.35 vol%), established by Health Canada (1995). In several European countries and the USA, the CO_2_ ALTER ranges from 1000 to 1200 ppm. The recommended indoor H_2_S concentration for residences is very low (maximum value = 106.5 ppb, 0.1 ppm; WHO, 2000) to avoid its unpleasant rotten-egg odour; this value is much below the H_2_S LOAEL (Lowest Observed Adverse Effect Level) (6.4 ppm; Dorman & Foster, 2016). The ISS study suggests that the ALTER values in Italian residences should be of 1000 ppm (0.1 vol%) for CO_2_ and 0.1 ppm for H_2_S (Settimo et al., 2016). These values refer to full comfort life conditions, considering the most vulnerable people (infants, children, pregnant women, elders), and they can be slightly exceeded without serious consequences for human health.

Many casualties and severe health problems have been caused worldwide by the inhalation of natural CO_2_ and/or H_2_S (Hansell & Oppenheimer, 2004; Lewis & Copley, 2015; Viveiros et al., 2016; Bustaffa et al., 2020; IVHHN, 2020; Nuvolone et al., 2020). The most affected countries are Italy, Japan, New Zealand but also Portugal in the Azores (Viveiros et al., 2015 and references therein). In particular, Rotorua city, built in a geothermal site of New Zealand, is severely exposed to endogenous gas hazard (Durand & Scott, 2003; Durand & Wilson, 2005). In Italy, at Vulcano (Aeolian Islands), two children and many small wild animals have been killed by CO_2_ emissions and tourists frequently report symptoms related to gas inhalation (Baubron et al., 1990; Carapezza et al., 2011). In the Lazio region of central Italy, several accidents have occurred to people because of natural gas inhalation (Chiodini et al., 2008): in 2000 two hunters died at Veiano; in 2011 a man died and other three were intoxicated at Lavinio (Barberi et al., 2019) and two persons died in an open-air SPA at Suio. Moreover, many animals have been killed by gas in nature reserves, such as at Tor Caldara, Solforata, Manziana (Carapezza, et al., 2010 and 2020a; Ranaldi et al., 2016). In addition, in the Metropolitan City of Rome gas blowouts from shallow drillings killed pets and birds, intoxicated people and led to the temporary evacuation of some houses (Barberi et al., 2007; Carapezza & Tarchini, 2007; Carapezza et al., 2020b).

**Fig. 1:**
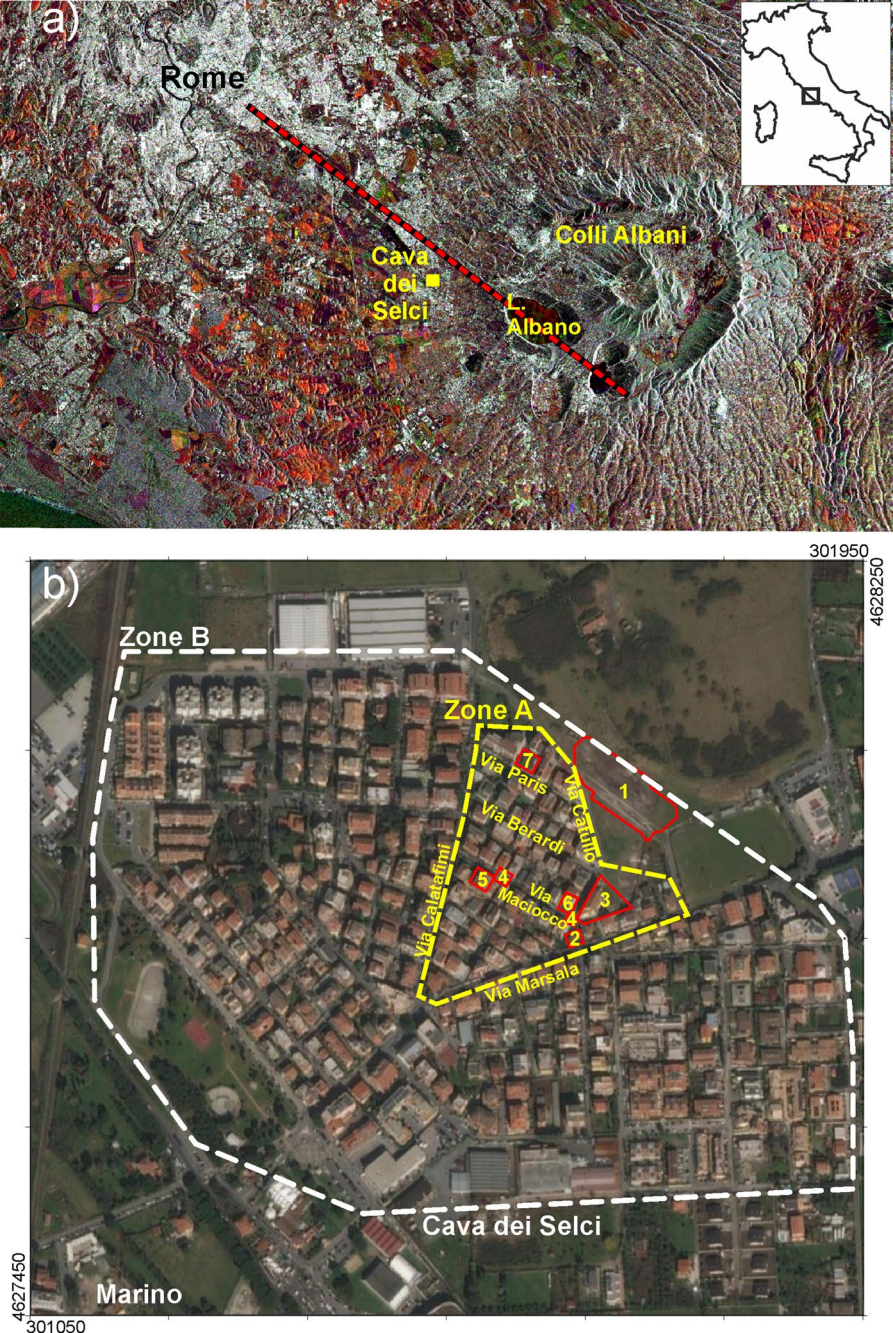
**a** Satellite image of Colli Albani volcano and Rome city, with location of Cava dei Selci; the dashed red line indicates the main NW-SE volcano-tectonic lineament (after Acocella & Funiciello, 2006). **b** The Cava dei Selci urban zone and its natural gas discharge (encircled with red line, no. 1). The other yellow numbers in red polygons indicate the sites of gas-related accidents. The white dotted line (zone B) is the perimeter of the area investigated for soil CO_2_ flux. The yellow dotted line (zone A) is the perimeter of the zone investigated for indoor air gas concentration. Coordinates are expressed in UTM WGS84 33 N

Cava dei Selci is a neighbourhood of Marino municipality, in the Metropolitan City of Rome, located at the northwest periphery of Colli Albani volcano (Fig. 1a). It hosts a former stone quarry particularly exploited in the XIX century, with a small depression that in rainy season hosts a stagnant water pool. This is the most important gas discharge of the Colli Albani volcano, where gas killed dozens of cows, sheep, pets and also a man lost his life nearby (Carapezza et al., 2003). The gas discharge of Cava dei Selci (no. 1 in Fig. 1b) has been geochemically monitored for over 20 years. In the highly emissive 2000 winter, the total CO_2_ output was estimated at 98 tonnes * day^−1^ from a 12,000 m^2^ area (Carapezza et al., 2003). In the following years, the diffuse soil CO_2_ flux has been regularly measured over a target area of 6350 m^2^, finding significant time variations from 3 to  ~ 25 tonnes * day^−1^, with oscillations (Carapezza et al., 2019). In 2007, the total diffuse soil H_2_S flux from the target area was estimated to 84 kg/day, while the simultaneous diffuse soil CO_2_ flux was 10.4 tonnes * day^−1^ (Carapezza, et al., 2010). Geochemical data demonstrate that H_2_S is the main cause of fatal incidents at Cava dei Selci discharge as it reaches, more frequently than CO_2_, immediately lethal air concentrations (> 450 ppm) at 25 cm from the ground (Carapezza, et al., 2010).

Starting from the 1960s, the neighbourhood of Cava dei Selci began to develop, and despite its high gas hazard, the zone near to the gas discharge was wildly urbanized in the period 1980–2000. Since 1999, a series of gas-related accidents and problems occurred showing that Cava dei Selci is one of the inhabited zones most exposed to endogenous gas hazard in the world.

The geochemical data on gas hazard were regularly presented to the Civil Protection Department of Lazio Region and Marino municipality. However, only after the 2008 and 2010 gas accidents occurred in the inhabited zone (see chapter 4), the problem was seriously considered by authorities. In 2010, the Epidemiology Department of Lazio was encharged to carry out a gas health effect study on Cava dei Selci population, whereas INGV was requested to expand the geochemical studies for a more precise gas hazard assessment of the area.

Results of this joint study on the natural gas emission and its health effects on exposed population are presented hereafter. In particular, we investigated whether there is an association between living near a natural gas discharge and mortality or emergency room visits (ERVs).

## Geological and geochemical overview

Cava dei Selci is sited within the Colli Albani volcanic complex, a Quaternary alkali-potassic volcano belonging to the Roman co-magmatic province of southern-central Italy (Washington, 1906) (Fig. 1a). Colli Albani volcanic activity began ~ 600 ka ago and continued up to at least 36 ka (Marra et al., 2016 and references therein). Lahars produced by floods from Albano crater lake occurred also in Roman times (IV century b.C., Funiciello et al., 2010). These mudflows generated a surficial impermeable layer, which covers a wide downslope area north of the Albano Lake (Funiciello et al., 2010 and references therein), including Cava dei Selci zone. Colli Albani is a quiescent volcano, and its periodic seismic crises, anomalous ground uplift, together with the magmatic imprint of the released cold gas, confirm that it may erupt again in the future (Carapezza, Barberi, et al., 2010; Marra et al., 2020; Trasatti et al., 2018). The most recent craters of Colli Albani cut the lake Albano floor and are aligned in a NW-SE direction (Anzidei et al., 2007), whose extension towards NW passes near Cava dei Selci gas discharge (Fig. 1a). This is an important tectonic lineament of Colli Albani (Acocella & Funiciello, 2006) and geochemical evidence indicates that, at present, it is still actively degassing (Carapezza et al., 2020b).

We analysed the structural lineaments of the northwest sector of Colli Albani, including Cava dei Selci, through field geological observations and photo-geological interpretation. The dominant lineaments are prevailingly NW-SE and WNW-ENE oriented, and secondarily N-S and NNE-SSW (Fig. S1 in Supplementary Material). At Cava dei Selci, the main orientations are NW-SE and NNW-SSE.

The gas emitted at Cava dei Selci, as in the other Colli Albani gas discharges, is dominated by CO_2_ (ca. 98 vol %) with ~ 1 vol % of H_2_S. It has a low temperature, and this has been explained by the extensive cold water circulation within the volcanic complex (Carapezza & Tarchini, 2007; Mazza & Capelli, 2010). The helium isotopic composition of Cava dei Selci gas (R/Ra = 1.23–1.54) indicates its deep magmatic origin (Carapezza & Tarchini, 2007; Martelli et al., 2004). The gas rising from depth encounters a buried Mesozoic calcareous formation, which hosts the main regional aquifer of Colli Albani (Mazza & Capelli, 2010). Here gas dissolves into the aquifer and mixes with CO_2_ generated by decarbonation reactions as indicated also by the carbon isotopic composition of the gas (Carapezza et al., 2021). Gas escapes to the surface along leaky faults, generating gas discharges in sites, like Cava dei Selci, where excavation removed the surficial impervious cover. Elsewhere gas pressurizes shallow confined aquifers, and gas blowouts may be generated by drillings (Carapezza, Barberi, et al., 2010 and 2020b).

## Geochemical study of the inhabited zone of Cava dei Selci

In 2010–2011, we carried out a detailed geochemical study of Cava dei Selci urban zone near the gas discharge aimed at assessing its gas hazard. The limits of the investigated zones are shown in Fig. 1b, where the sites of the gas-related accidents, which will be addressed in the following, are also indicated.

### Materials and methods

Soil CO_2_ flux measurements were carried out with the accumulation chamber method (Chiodini et al., 1998) by a West Systems portable fluxmeter. The device is equipped with an IR Licor-Li820 detector for CO_2_ (single-beam dual wavelength NDIR; range 0–2 vol%; accuracy 3%). The measurements were always carried out in dry and stable weather conditions to reduce the possible atmospheric influence on soil CO_2_ flux (Carapezza & Granieri, 2004).

The soil CO_2_ flux maps were obtained in ArcGIS 10.6 by conditional Gaussian geostatistical simulation of the simple Kriging model. The soil CO_2_ flux classes (statistical levels) were obtained by graphical investigation of the normal probability plot of the flux data (Yusta et al., 1998).

The soil concentration of CO_2_ and H_2_S was measured using a steel probe inserted in the ground at 50 cm depth and connected by a silicon tube to a portable multi-gas device (Draeger X-am 7000). The device was equipped with an IR CO_2_ detector (scale: 0–100 vol%; accuracy: 3% of readings) and electrochemical cells for H_2_S (0–1000 ppm; accuracy: 5% of readings). The same device was used to measure air CO_2_ and H_2_S concentration. Electrolytic cells with different scales were used for H_2_S concentration monitoring: 0–100, 0–500, 0–1000, 0–2500 ppm with a 5% accuracy of readings and data acquisition frequency of 1 min. Air concentration measurements were carried out generally at 15 cm height from the ground; if gas concentration was dangerous, measurements were repeated also at 50, 100 and 150 cm height.

To mitigate the gas risk during the fieldwork, in case of high acid gas concentration the members of our team wore a protective gas mask. A light compressed-air breathing apparatus was used in case of low indoor oxygen concentration.

### Soil CO_2_ flux survey and in-soil gas concentration

**Fig. 2:**
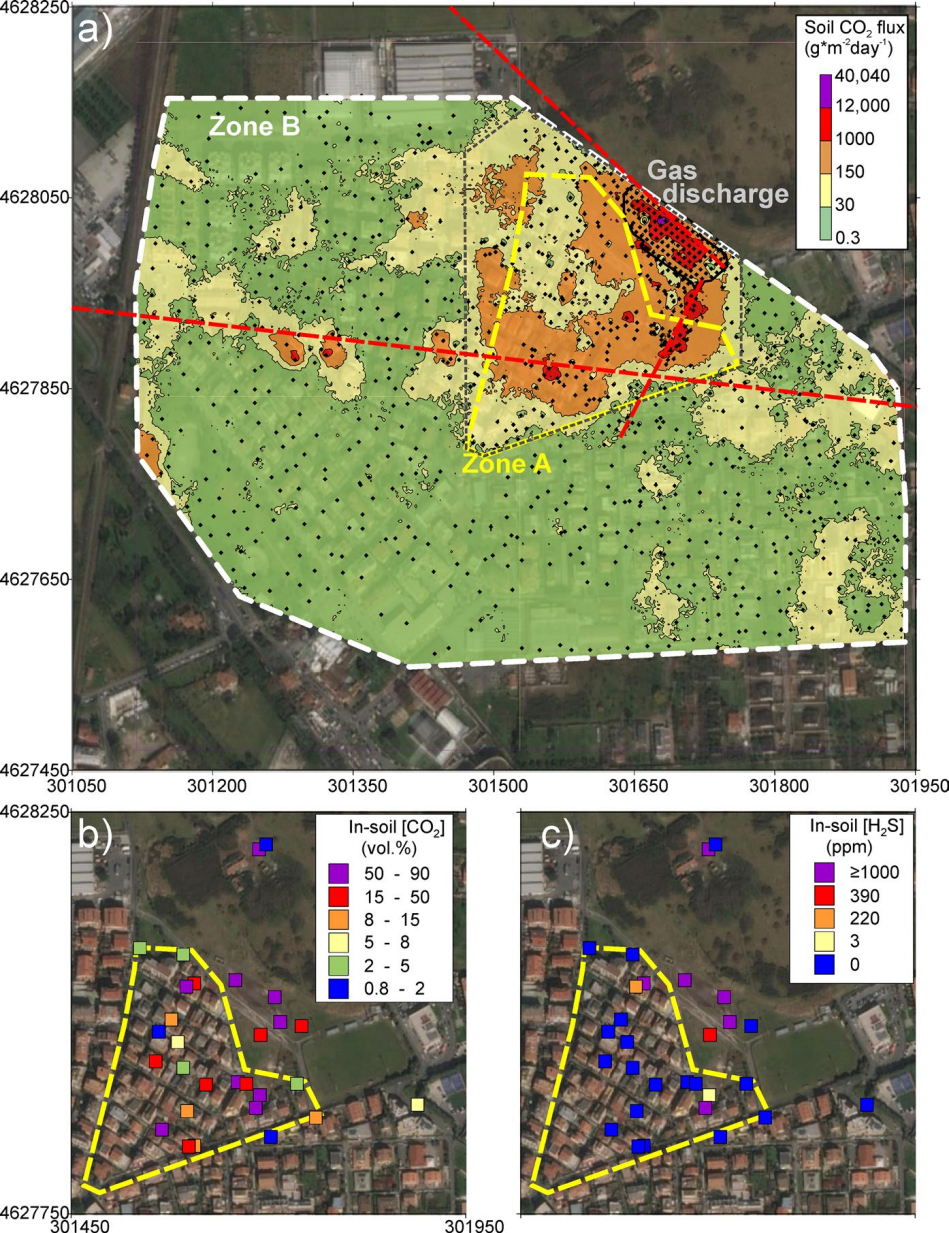
Results of the geochemical survey of February–March 2011. **a** Soil CO_2_ flux map of Cava dei Selci (black dots = survey points). (**b**) and (**c**) In-soil concentration at 50 cm depth of CO_2_ and H_2_S, respectively. Satellite base map from Google Earth; coordinates in UTM WGS84 33 N

A wide survey of soil CO_2_ flux was carried out in February–March 2011 over an area of 394,720 m^2^ (dataset in Carapezza et al., 2020c). All the open spaces and house gardens of Cava dei Selci urban zone (zones A and B in Fig. 1b), and the gas discharge area, were investigated with 1143 measurements. The total diffuse soil CO_2_ flux was estimated to 32.12 tonnes * day^−1^. The related map (Fig. 2a) shows that the gas discharge is the most emissive area, with a large NW-SE trending anomaly (average soil CO_2_ flux = 1638 g * m^−2^ day^−1^). Here, a total soil CO_2_ output of 7.8 tonnes * day^−1^ was estimated from only 5800 m^2^. The soil CO_2_ flux map reveals also the presence of a wide anomalous zone extending from the gas discharge area to the zone A (black dotted line in Fig. 2a). Excluding the gas discharge, this zone covers an area of 68,380 m^2^ and releases 15.92 tonnes * day^−1^ of CO_2_.

It is important to note that soil CO_2_ fluxes measured in house gardens of zone A (yellow perimeter in Fig. 2a) were almost all much higher than the local biological threshold due to soil respiration and estimated at 30 g  *  m^−2^ day^−1^ (Carapezza et al., 2003). The average soil CO_2_ flux is quite anomalous (202 g * m^−2^ day^−1^), and this indicates that, besides the gas discharge, also in the urbanized zone A there is a high and potentially hazardous CO_2_ release from the soil. In this zone, also the in-soil gas concentration is very high, with CO_2_ values ranging from 5 to 90 vol% in 15 out of 20 sites (Fig. 2b), and H_2_S ranging from 220 to ≥ 1000 ppm in three sites (Fig. 2c).

Conversely, most of zone B is characterized by soil CO_2_ fluxes within the local background class (green in Fig. 2a). The only exceptions are the area bordering the western side of zone A and a narrow anomalous WNW-ESE-oriented band extending across the entire investigated area. The orientation of the main gas-releasing zones (red dashed lines in Fig. 2a) corresponds to the main tectonic alignments of the area (Fig. S1 in Supplementary Material), confirming the tectonic control on deep gas release.

### Indoor and outdoor air gas concentration measurements

In 2010, along the main roads of zone A (Fig. 1b), 138 residential and not residential (garages, cellars, basements) rooms were investigated with 805 indoor punctual measurements of air CO_2_ and H_2_S concentration. Air gas concentration was measured also in 40 nearby outdoor sites. Results are summarized in Table 1, where the soil CO_2_ flux values recorded at the same time in the house gardens are also reported (the related dataset can be found in Carapezza et al., 2020c).

**Table 1:** Indoor and outdoor air gas concentration and soil CO_2_ flux—2010 survey at Cava dei Selci

Road	Location		Measure	Air gas concentration*				Soil CO_2_ flux		
(Location in Fig. 1b)	Indoor	Outdoor	no.	CO_2_ (vol%)		H_2_S (ppm)		(g*m^−2^ day^−1^)		
				Max	Avg	Max	Avg	Min	Avg	Max
*Calatafimi*	40	5	123							
Residential			79	1.0	0.64	0	0			
Non-residential			34	6.0	1.12	31	1.6			
Outdoor			10	26	5.0	≥ 1000	137	10.0	47.5	123.9
*Catullo*	4	2	27							
Residential			11	4.2	0.55	48	6.6			
Non-residential			10	1.0	0.16	0	0			
Outdoor			6	11	2.0	24	4.8	17.1	344.1	2051.3
*Berardi*	19	7	166							
Residential			109	22.9	0.47	30	0.3			
Non-residential			33	18	1.79	0	0			
Outdoor			24	1.8	0.21	0	0	11.5	359.1	5722.1
*Maciocco*	26	8	173							
Residential			97	15.5	0.66	7.0	0.2			
Non-residential			25	4.8	0.98	7.1	71			
Outdoor			51	42	3.96	≥ 1000	65	17.1	120.2	870.4
*Marsala*	28	7	171							
Residential			121	6.2	0.19	273	3.7			
Non-residential			34	12	1.10	0	0			
Outdoor			16	4	0.74	0	0	7.3	71.1	865.8
*Paris*	21	11	185							
Residential			101	8.6	0.34	90	1.3			
Non-residential			44	72	4.50	≥ 500	51			
Outdoor			40	29	2.33	≥ 1000	51	15.0	77.8	261.7
Total	*138*	*40*	*845*							

*Minimum concentration: H_2_S = 0, CO_2_ = 388 ppm (i.e. air value in 2010; NOAA, 2020)

Over 800 measurements of indoor gas concentrations show average values of CO_2_ between 0.19 and 0.64 vol%, and of H_2_S between 0 and 6.6 ppm. In all investigated sites soil CO_2_ flux varies from slightly anomalous (47.5 g * m^−2^ day^−1^) to strongly anomalous (359.1 g * m^−2^ day^−1^) indicating the endogenous origin of the gas.

**Fig. 3:**
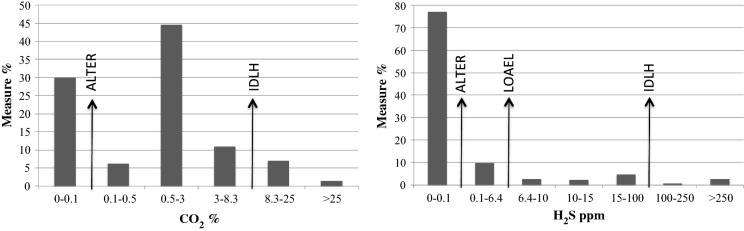
Frequency histograms of indoor CO_2_ and H_2_S concentration in the urban zone A (see the introduction chapter for the definition of the gas hazard thresholds)

Indoor results are presented in the histograms of Fig. 3 where the gas concentration hazard thresholds are also indicated. Inside 70% of the residences, CO_2_ concentration was above 0.1 vol% (i.e. the ALTER recommended by Italian Health Service); in 8% it was above IDLH (8.3 vol%) up to 22.9 vol% in residential and up to 72 vol% in non-residential rooms. Inside 23% of the residences H_2_S concentration was above ALTER (0.1 ppm), with 14% above LOAEL (6.4 ppm) and 4% above the IDLH level (100 ppm), with a maximum of 273 ppm in residential and ≥ 500 ppm (upper limit of the sensor) in non-residential rooms. Hazardous air gas concentrations were found also outdoor, up to lethal values: 42 vol% of CO_2_ and ≥ 1000 ppm of H_2_S (upper limit of the sensor) in an airhole of a house.

## Accidents and problems related to endogenous gas release at Cava dei Selci

Many Cava dei Selci houses of zone A are located at a few hundred metres distance from the gas discharge (Fig. 1b). Via Maciocco is the road where most accidents and problems related to the emission of endogenous gas have occurred. The gas-related accidents are listed hereafter with the same numbers used in Fig. 1b for their location (dataset in Carapezza et al., 2020c).

1. Cava dei Selci gas discharge: death by gas inhalation of 29 cows on 18 September 1999 and of 6 sheep on 11 March 2000. Dead cats and wild small mammals are frequently found (Carapezza et al., 2003).
2. Via Marsala: death by gas asphyxiation of a man, 30 December 2000 (Carapezza et al., 2003).
3. Via Maciocco: gas blowouts from shallow drillings, 16–19 May 2008; definitive access interdiction to the ground floor of a nearby pilotis-building because of hazardous air gas concentration (Carapezza et al., 2010b).
4. Via Maciocco: three sudden collapses of road sectors in 2008, 2012 and 2019 (sub-chapter 4.1).
5. Via Maciocco: evacuation of three flats because of hazardous indoor gas concentration, 24 February 2010 (sub-chapter 4.2).
6. Via Maciocco: evacuation of one flat because of hazardous indoor gas concentration, 20 March 2018 (sub-chapter 4.4).
7. Via Paris: diseases of residents because of high indoor gas concentration, 2008–2011 (sub-chapter 4.3).
8. In the following, we describe the main gas-related accidents occurred in Cava dei Selci-zone A, after the May 2008 gas blowouts, summarizing the scientific investigations carried out to assess the related hazard.

### *The *via* Maciocco road collapses*

**Fig. 4:**
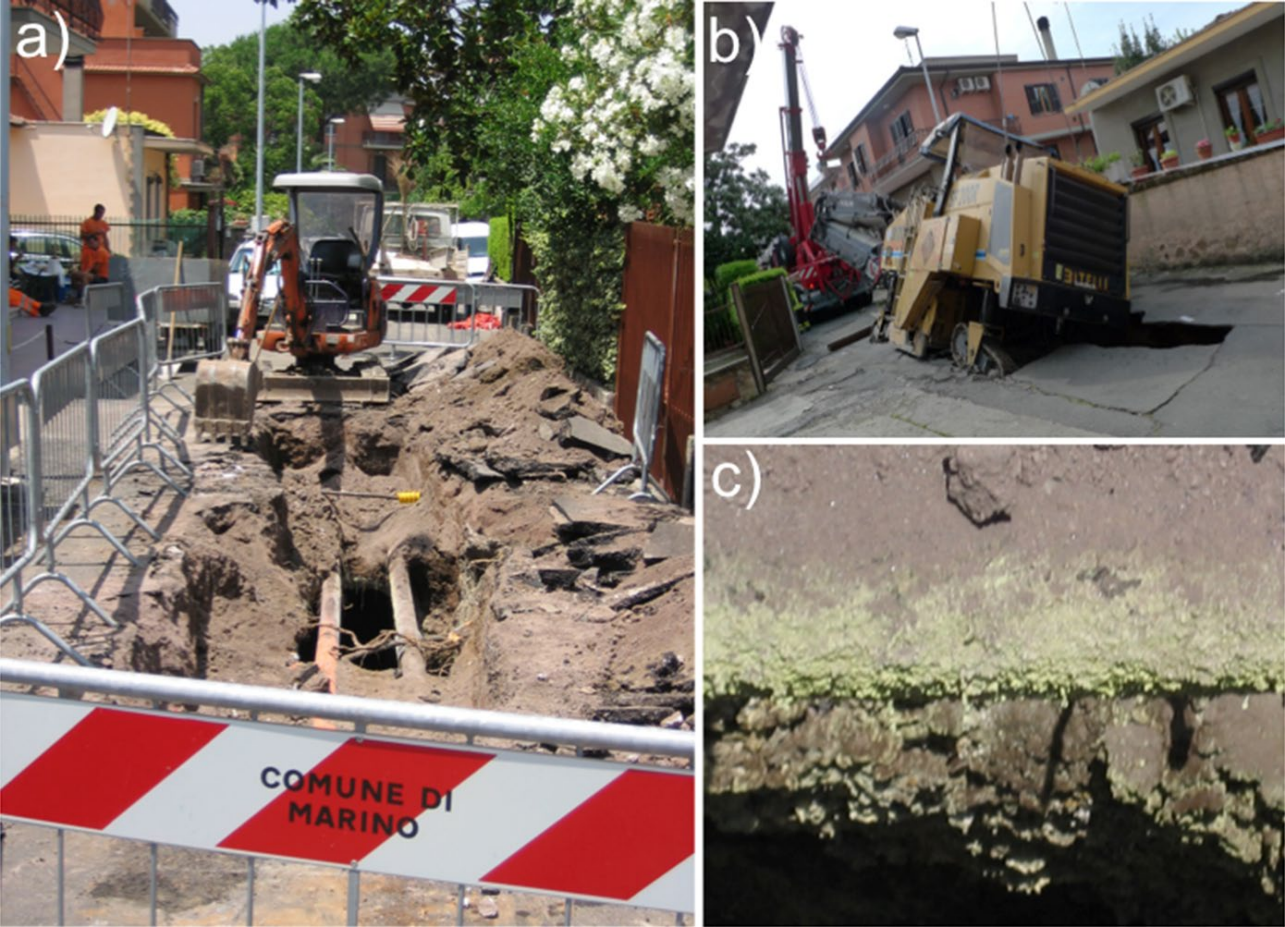
**a** and **b** The 2008 and 2012 collapses of two sectors of via Maciocco. **c** Sulphur layer on the channel wall of (**a**)

In June 2008, a portion of via Maciocco (site no. 4 in Fig. 1b) suddenly collapsed during the transit of a heavy truck, showing that the road had been built covering a pre-existing water channel where endogenous gas was released (Fig. 4a). Actually, on the channel walls, a layer of sulphur sublimate was observed (Fig. 4c). The gas released from the collapsed portion of the road created irritation to the respiratory tract of most residents, who were also disturbed by the H_2_S odour. It took three days to restore the road and the problem of the gas emission underneath it remained unsolved. On May 2012 (Fig. 4b) and December 2019, two new similar collapses occurred again in other sectors of via Maciocco.

Covering gas-releasing ditches to build roads was unfortunately a bad common practice at Cava dei Selci. Another example is the ditch bordering to the NW the gas discharge, where a CO_2_ output of 2.5 tonnes * day^−1^ from only ~ 60 m^2^ had been measured by Carapezza et al. (2003). Presently a road covers this ditch (Via Catullo in Fig. 1b).

### *The february 2010 accident in *via* Maciocco*

On 24 February 2010, the Fire Brigade, alerted by a woman who had suffered an illness inside her house, inspected two contiguous flats at via Maciocco (designed here as 1A and 1B; site no. 5 in Fig. 1b). They found dangerous indoor air gas concentration, particularly inside the basement floor and ordered the immediate evacuation of the two flats. Since the following day, we started a geochemical investigation to assess the health gas hazard of the site. Both flats were made of a ground floor hosting a living room, a bedroom and a bathroom, and of a basement floor with a kitchen, a bathroom and a catchall. Indoor air gas concentration was measured five times, from 25 February to 6 March 2010, in all ground floor and basement rooms (with closed openings) and in the common garage open space; results are reported in Table 2. At the ground floor, slightly anomalous air gas concentration values were found: maximum 2 ppm of H_2_S and 1.4 vol% of CO_2_ in the living room of flat 1A; maximum 3 ppm of H_2_S and 2.2 vol% of CO_2_ in the bathroom of flat 1B. High and lethal air gas concentrations were found in the basement. In the kitchen of flat 1A we found: H_2_S ≥ 100 ppm (upper limit of the device) and CO_2_ 14.5 vol%. In the catchall and kitchen of flat 1B we found: H_2_S ≥ 100 ppm and CO_2_ = 24 vol% (Table 2; data not considered in Table 1).

**Table 2:** Air concentration of CO_2_ and H_2_S measured in February–March 2010 inside two evacuated flats of via Maciocco (at 15 cm from the ground, if not differently indicated)

	25 February		26 February		2 March		3 March	6 March
*Flat 1A-ground floor*	CO_2_ vol%	H_2_S ppm	CO_2_ vol%	H_2_S ppm	CO_2_ vol%	H_2_S ppm	CO_2_ vol%	CO_2_ vol%
Living room	0	2	0.6	0	0	0	0	1.4
Bedroom	0	0	0.4	0	n.d	n.d	n.d	n.d
Bathroom	0	0	0.4	0	0	0	1.0	0.6
Shower drainhole	1.0	0	0.6	0	0.4	0	0.6	n.d
*Flat 1A-basement*								
Kitchen	2.0	60	12.4	33	12.8	≥ 100	14.5	2.0
Kitchen (90 cm)	1.0	8	3.4	8	7.7	≥ 100	8.2	1.2
Window on garage (1 m)	0.2	4	1.0	3	6.0	82	6.8	n.d
*Flat 1B-ground floor*								
Living room	0	0	1.2	2	0.6	0	1.4	
Bedroom	0	0	1.4	0	0	0	2.0	
Bathroom	0.6	2	0.8	2	1.0	0	2.2	
Shower drainhole	0.6	3	0.8	3	1.0	0	2.0	
*Flat 1B-basement*								
Kitchen	1.2	7	16.4	≥ 100	12.0	15	10.0	
Bathroom	1.6	0	9.8	10	10.4	7	13.0	
Catchall	3.8	14	24.0	≥ 100	12.0	27	14.0	
*Garage space*								
Well no. 1 (wellhead, 1 m)*	99	3560	n.d	n.d	6.8	52	n.d	n.d
Well no. 2 (outside wellhead)*	60	2420	n.d	n.d	1.8	32	3.8	n.d
Cockpit	3.0	64	2.4	4	3.6	≥ 100	3.2	
Cockpit	5.4	232	1.4	12	7.4	≥ 100	2.8	
Cockpit	1.2	8	1.2	8	0.6	15	2.0	
Cockpit	2.2	48	1.8	15	3.4	20	1.6	
Lift door	2.6	26	2.6	21	n.d	n.d	4.4	
Inside garage	4.2	0.6	4.2	0.6	n.d	n.d	n.d	
Inside garage	2.0	4	n.d	n.d	n.d	n.d	1.0	
Inside garage	5.2	11	n.d	n.d	n.d	n.d	n.d	

CO_2_ values in excess to the normal air concentration (0 = 388 ppm in 2010; NOAA, 2020); n.d = access not possible; *[H_2_S] by Draeger tubes

This high indoor gas concentration in both flats was due to the presence of two water wells in the basement open garage. These wells intercepted the shallow gas pressurized aquifer, which produced the 2008 nearby gas blowouts (point 3 in Fig. 1b). Within and near their wellheads, we measured very high gas concentrations up to 99 vol. % of CO_2_ and 3560 ppm of H_2_S (Table 2). Consequently, the Fire Brigade interdicted the access also to this garage area.

**Fig. 5:**
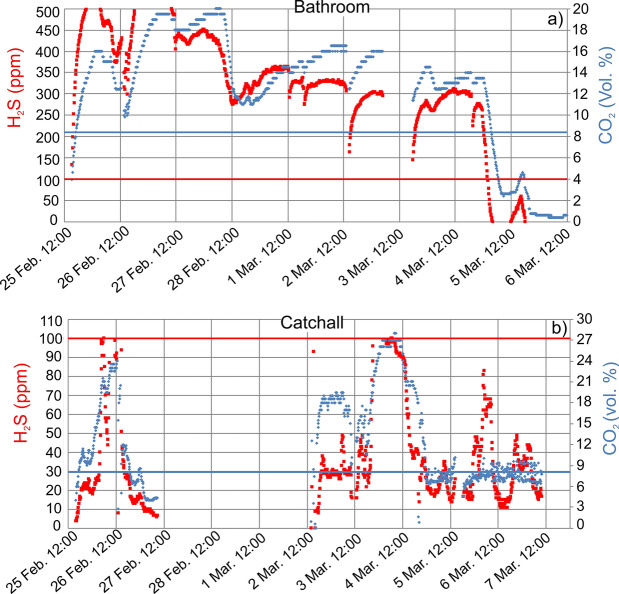
Indoor continuous monitoring of CO_2_ and H_2_S in the evacuated flats of Via Maciocco from 25 February to 7 March 2010. **a** 1A basement bathroom. **b** 1B basement catchall. Lack of data corresponds to electric blackout. Horizontal lines indicate the IDLH level for CO_2_ (blue) and H_2_S (red). The H_2_S upper detection limit was 500 ppm in (**a**) and 100 ppm in (**b**)

Since 25 February 2010, we continuously monitored for nearly ten days the indoor CO_2_ and H_2_S concentration, in the basement bathroom of flat 1A and in the catchall of flat 1B. In the bathroom (Fig. 5a) the concentration of both gases remained almost always above the IDLH levels, up to immediately lethal values (H_2_S ≥ 500 ppm, upper limit of the device; CO_2_ = 20 vol%). This is the site where the woman suffered the illness. In the catchall, the air CO_2_ concentration remained long above IDLH level up to 27.0 vol%, whereas H_2_S reached 100 ppm in two over seven nights (Fig. 5b).

On 4 and 6 March, lethal air CO_2_ values (20.6–22.0 vol%) were found inside the basement kitchen of the contiguous twin 1C flat. Gas was rising from the basement to the ground floor, where a concentration of 2.2 vol% was measured at 50 cm height in the bedroom. Such a concentration in the air breathed by residents during night explains the daily illness with dizziness and headache suffered at their awakening. Consequently the Fire Brigade evacuated also this flat.

**Fig. 6:**
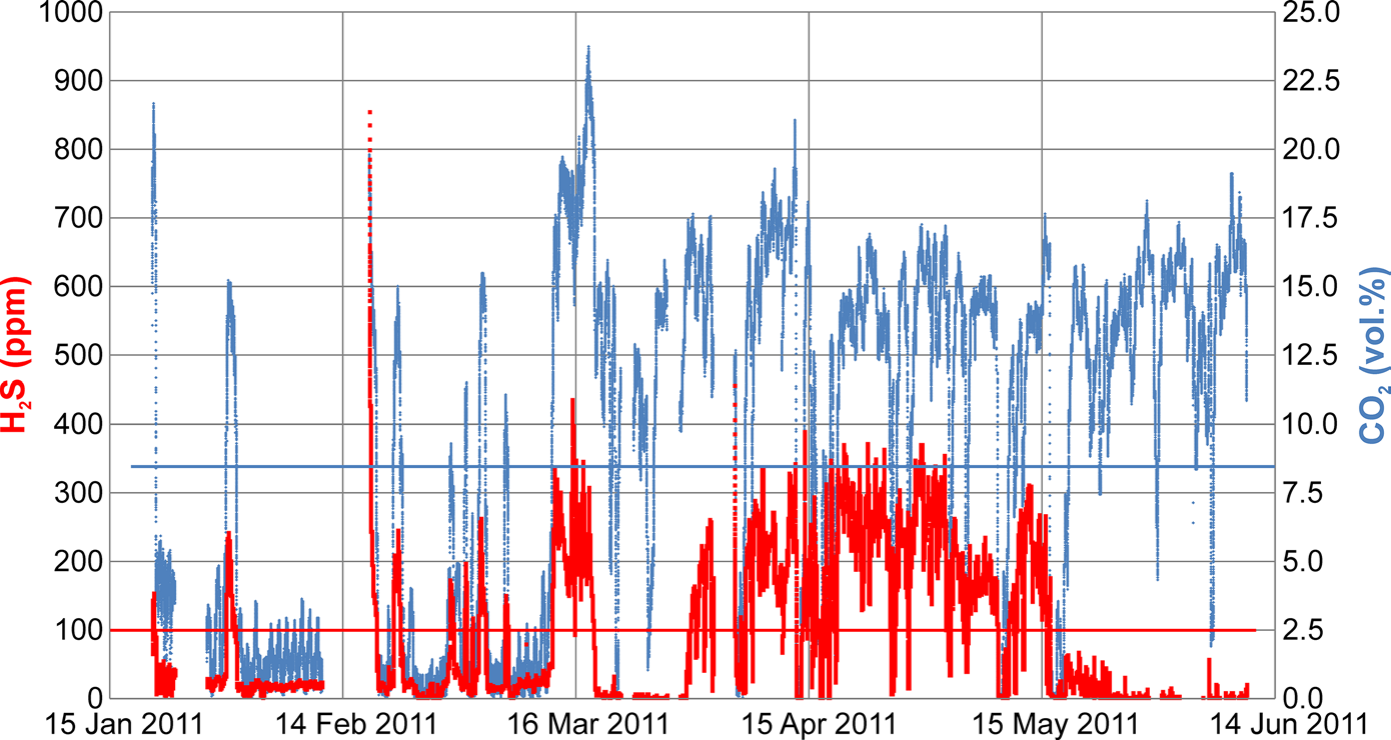
Indoor continuous monitoring of air CO_2_ and H_2_S concentration in the 1C basement kitchen from January to June 2011. The horizontal lines indicate the IDLH level for CO_2_ (light blue) and H_2_S (red). The H_2_S upper detection limit was 2500 ppm

In addition, from January to June 2011, we continuously monitored the indoor CO_2_ and H_2_S concentration in the basement kitchen of the evacuated 1C flat (Fig. 6). Results show that during winter months, CO_2_ and H_2_S had medium-low average concentrations (average values = 5.6 vol% and 59.5 ppm, respectively) with few lethal peaks (up to 23.8 vol% and 854.8 ppm) and exceeded IDLH in 27% and 21% of the CO_2_ and H_2_S measurements, respectively. In springtime, 83.46% of CO_2_ concentrations were over IDLH with an average value of 12.4 vol% and a maximum value of 21.1 vol%. H_2_S had a similar behaviour (average 116.5 ppm; maximum 456.7 ppm) but with a strong decrease after mid-May (Fig. 6). During spring, 50.3% of H_2_S values were over IDLH. This behaviour can be explained by considering that the area received heavy rain during winter. The surficial aquifer was therefore enriched in fresh cold meteoric water, which dissolved more gas with respect to spring–summer time, with consequent decrease in its release to the atmosphere.

### *Gas hazard in *via* Paris*

In 2010, we investigated another Cava dei Selci house located in via Paris (site no. 7, Fig. 1b). Here the resident family was suffering diseases, e.g., dizziness and headache, likely due to gas inhalation. In particular, a young man was suffering chronic prostration, attributed by physicians to depression. This is a single-floor old edifice, with a crawl space below the house. A CO_2_ concentration of 100 vol%, with H_2_S ≥ 500 ppm, was measured within this crawl space, and anomalous gas concentrations were found outside the entrance door and the bedroom window (Fig. S3 in Supplementary Materials).

**Fig. 7:**
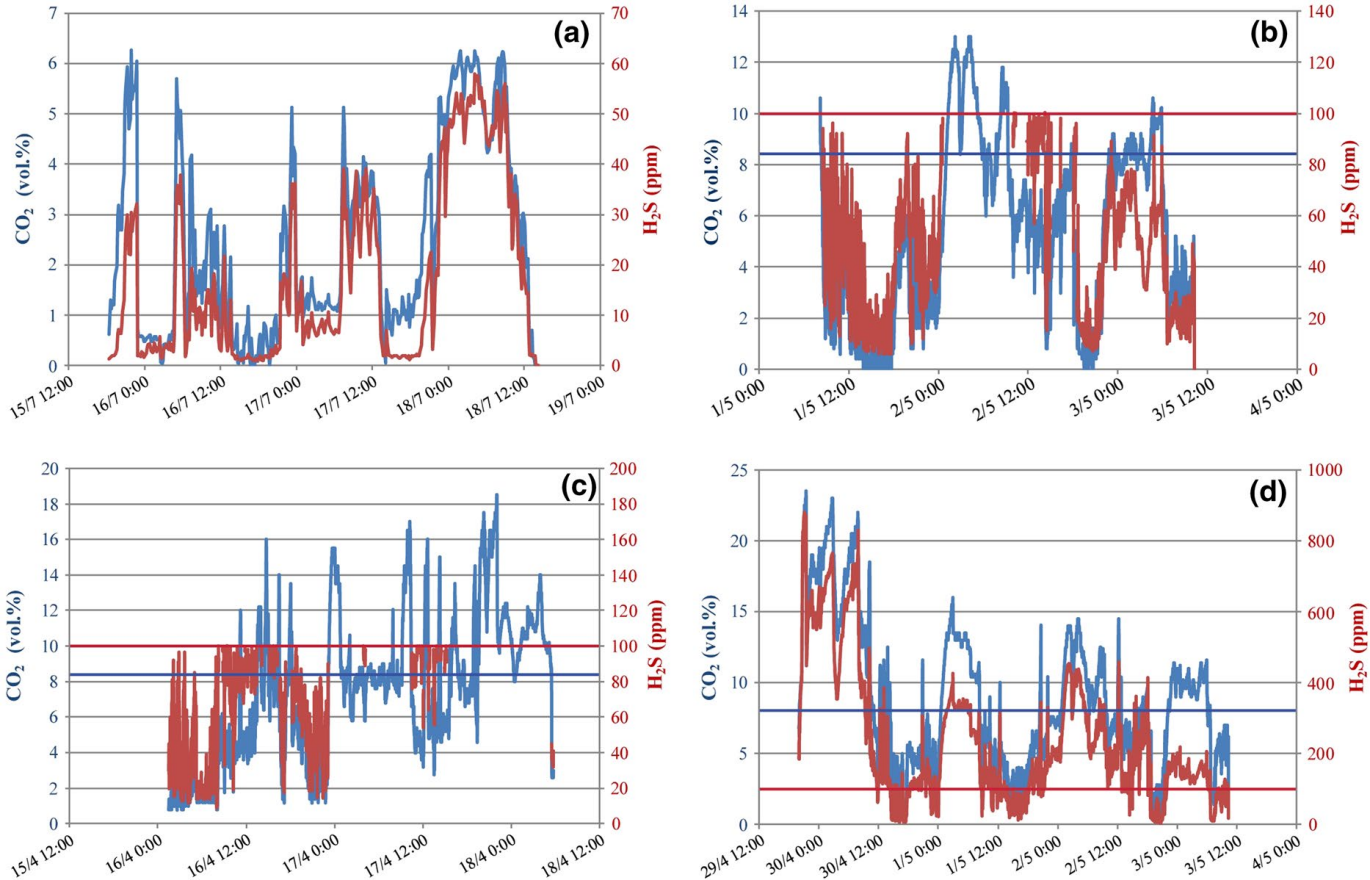
Indoor continuous recording of CO_2_ and H_2_S concentration at via Paris. Living room: 15–19 July 2010 (**a**) and 1–3 May 2011 (**b**). Bathroom: 15–18 April 2011 (**c**) and 29 April-3 May 2011 (**d**). Measure frequency: 10’ in (**a**); 1’ in (**b**), (**c**), (**d**). The horizontal lines indicate the IDLH level for CO_2_ (blue) and H_2_S (red)

Continuous recordings of indoor air gas concentration carried out in 2010 and 2011 showed that the living room and the bathroom were severely exposed to gas hazard. In the living room, CO_2_ and H_2_S repeatedly overpassed their IDLH levels, up to 13 vol% and ≥ 100 ppm, respectively (Fig. 7a, b). In the bathroom, CO_2_ reached a maximum of 18.5 vol%, whereas H_2_S remained long at 100 ppm (the upper limit of the used device) (Fig. 7c). Therefore, air H_2_S concentration in the bathroom was monitored again, increasing to 2500 ppm the device upper limit (Fig. 7d). The H_2_S concentration almost continuously exceeded 100 ppm with a maximum of 880 ppm; the CO_2_ concentration showed simultaneous variations with a maximum of 23 vol% (Fig. 7d).

In the two bedrooms, gas concentration was significantly above the respective ALTER levels (up to 1.5 and 3.0 vol% of CO_2_ and 12 ppm of H_2_S). The long-term exposition to these concentrations may explain the diseases suffered by the residents. Some gas-proof remediation works were carried out in 2011 and reduced significantly the gas emission with consequent health benefits for the residents. However, we recommended residents to install a forced air ventilation system.

### *The March 2018 accident in *via* Maciocco*

**Fig. 8:**
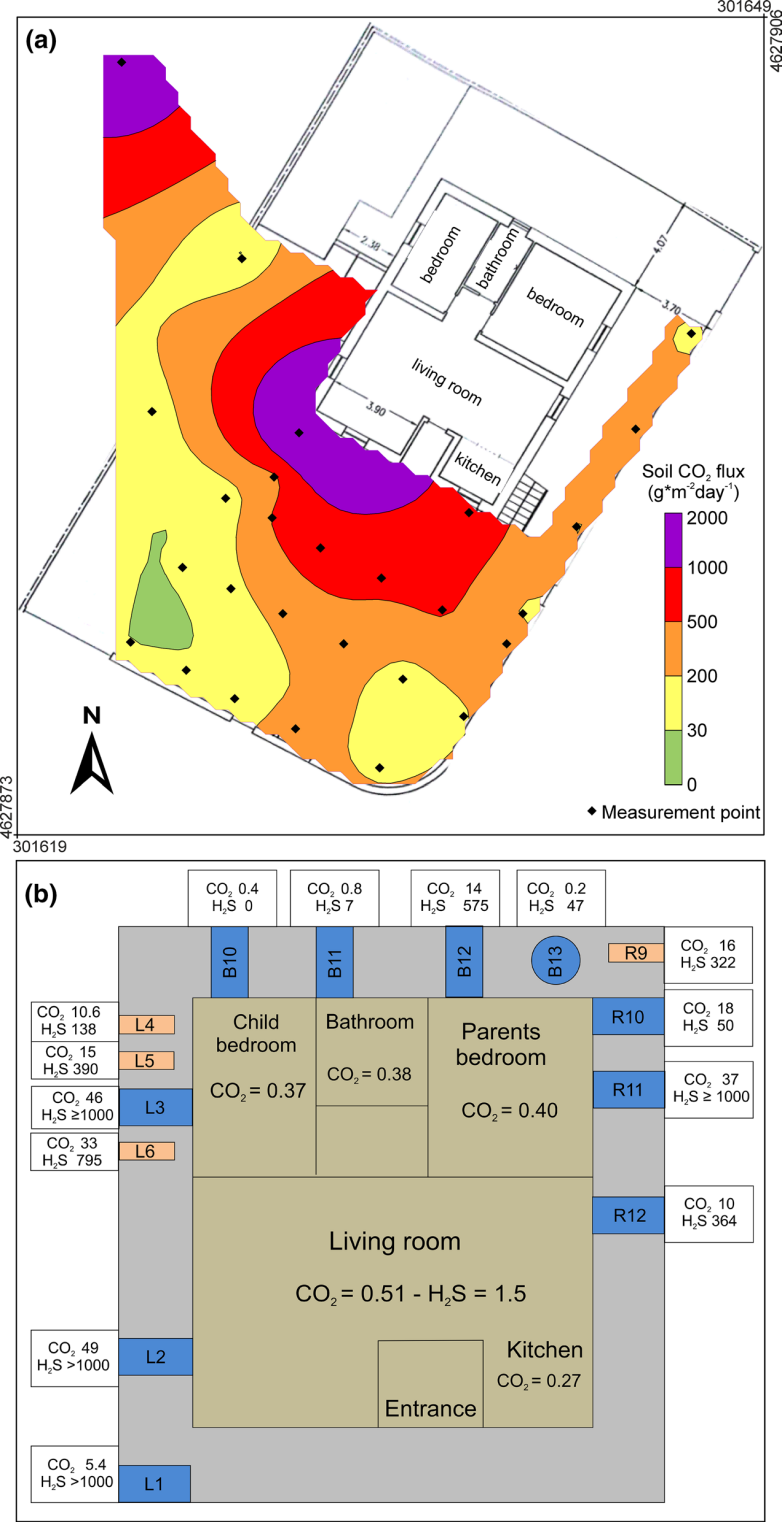
**a** Soil CO_2_ flux map of the courtyard of Via Maciocco house evacuated in March 2018 (site no. 6, Fig. 1b). **b** Schematic map of the evacuated house with indication of the maximum values of CO_2_ (vol%) and H_2_S (ppm) measured indoor and outdoor. Letters B, L, R indicate air vents (rectangles) and a drain grate (circle)

On March 2018, the Fire Brigade intervened again in a via Maciocco house (site no. 6 in Fig. 1b). At the ground level of the house, there was an evident swelling of the living room floor, which had created concern in the resident family. The house had been evacuated and parking and transit interdicted in the courtyard of the building. An essay showed the presence of a gas-proof sheath, covering a layer of loose material with evidence of caving and sulphur incrustations (Fig. S2a in Supplementary Materials). Outside the house entrance, the deformation had detached some tiles (Fig. S2b in Supplementary Materials) and anomalous CO_2_ flux was measured on these detachments. A weak swelling with associated fractures was observed also in the courtyard of the adjacent building (Fig. S2c in Supplementary Materials) where a very high soil CO_2_ flux was measured (see Fig. 8a).

On 23 March 2018, a soil CO_2_ flux survey was carried out in the garden of the evacuated house and in the courtyard of the adjacent building, with 27 measurements over a surface of 300 m^2^. Flux values ranged from 9.5 to 2000 g * m^−2^ day^−1^ with an average of 378 g * m^−2^ day^−1^. The related gas flux map (Fig. 8a) shows that the area was almost entirely interested by an anomalous soil CO_2_ release (> 30 g * m^−2^ day^−1^), with the highest flux values distributed along the perimetric belt of the house.

The air concentration of CO_2_ and H_2_S was daily measured along four days inside all rooms of the evacuated house. Measurements were made also near electrical sockets, hydraulic dumping and on small fractures of the floor. In the living room, air CO_2_ concentration slightly increased from 20 to 23 March from 0.2 up to 0.51 vol%. Here 1.5 ppm of H_2_S was found, whereas in all other rooms H_2_S concentration was null. Continuous monitoring (3 days) of gas over the beds displayed CO_2_ values in the same range (maximum 0.37 vol%), whereas H_2_S concentration resulted always null.

Very high gas concentration, up to 49 vol% of CO_2_ and ≥ 1000 ppm of H_2_S, was found outdoor, in correspondence of air vents located at the base of the external walls of the house (Fig. 8b). This is indicative of an intense degassing underneath the house, as indicated also by the increase of soil CO_2_ flux towards it (Fig. 8a).

Considering such high outdoor values, the air gas concentration was continuously measured from 1 to 4 days, at various heights, outside the main openings of the living room (entrance door and window) and of the child bedroom (window). The most anomalous values, up to over 5000 ppm of CO_2_ and 15 ppm of H_2_S, were found at 50 cm height outside the two investigated windows. Lower but still anomalous values (CO_2_ = 2520–3115 ppm; H_2_S = 8–9 ppm) were found in the same sites at 125 cm height (Table 3).

**Table 3:** Main results of continuous monitoring of outdoor air concentration (in ppm) of CO_2_ and H_2_S near house openings, carried out in March 2018

	Entrance door (3 days; height 125 cm)		Living room window (1 day; height 50 cm)		Living room window (4 days; height 125 cm)		Child bedroom window (1 day; height 50 cm)		Child bedroom window (2.5 days; height 125 cm)	
	CO_2_	H_2_S	CO_2_	H_2_S	CO_2_	H_2_S	CO_2_	H_2_S	CO_2_	H_2_S
Minimum	0	0	0	0	0	0	1069	0	0	0
Maximum	1943	8	5160	15	2520	8	5128	6	3115	9
Average	822	2	1784	3	900	1	1469	2	1026	2

CO_2_ values in excess to the normal air concentration (0 = 406 ppm in 2018; NOAA, 2020)

Results indicate that around the investigated house there is an anomalous gas release from the soil. The gas may also penetrate inside the house through openings, increasing the indoor hazard. The high outdoor gas concentration measured at 50 cm height indicates that in the external part of the house there is a potential gas hazard for children and pets. Results show that a large quantity of gas accumulated underneath the house floor within the highly porous loose material. It seems logical to infer that the floor swelling was caused by the gas pressure on the gas-proof sheath that fortunately resisted without breaking, so preventing a hazardous indoor gas release.

## Epidemiologic study of mortality and emergency room admissions of Cava dei Selci residents

The exposure to high concentration of CO_2_ and H_2_S can cause fatalities from asphyxiation and increase respiratory, cardiovascular and nervous diseases (Bates et al., 2002; Hansell et al., 2004). Considering the values of air gas concentration found at Cava dei Selci, a retrospective cohort study was carried out in 2010 to assess whether long-term gas exposure increased mortality and emergency hospital admissions in the resident population.

### Cohort enrolment

Using the Municipality Registries, we enrolled all residents (53,872 persons) living in Marino municipality from 1 January 1996 to 31 December 2008. Vital status (alive, dead, migrant) and the residence address at the beginning of the follow-up for each participant were collected and the baseline addresses were geocoded using the ArcGIS software. The follow-up for vital status (1996–2008) was carried out through record linkage with the regional mortality database, which includes all deaths of the resident population. A socio-economic position index was assigned to each resident based on the residence census block. This index was built using a factor analysis and taking information about educational level, job, immigrants and family size from the 2001 census. It was structured into five categories (high, middle-high, medium, middle-low and low) on quantile distribution of the population of the Lazio region (Cesaroni et al., 2006) (Table 4).

**Table 4:** Characteristics of the cohort by study zone, municipality of Marino (1996–2008). *Data * *source*: Municipality Register, City of Marino, Rome

Residents		Municipality of Marino							
		Reference zone		Zone B		Zone A		Total	
		no.	%	no.	%	no.	%	no.	%
Total		49,588	100	2889	100	1132	100	53,609	100
Gender	Men	24,168	48.7	1457	50.4	553	48.9	26,178	48.8
	Women	25,420	51.3	1432	49.6	579	51.1	27,431	51.2
Age class at recruitment	0	5793	11.7	346	12.0	149	13.2	6288	11.7
	1–14	5365	10.8	311	10.8	113	10.0	5789	10.8
	15–44	23,525	47.4	1427	49.4	605	53.4	25,557	47.7
	45–64	9684	19.5	524	18.1	182	16.1	10,390	19.4
	65 +	5221	10.5	281	9.7	83	7.3	5585	10.4
Socio-economic position	High	8024	16.2	0	0.0	0	0.0	8024	15.0
	Middle-high	12,080	24.4	493	17.1	0	0.0	12,573	23.5
	Medium	9998	20.2	202	7.0	60	5.3	10,260	19.1
	Middle-low	5462	11.0	451	15.6	545	48.1	6458	12.0
	Low	7250	14.6	1370	47.4	396	35.0	9016	16.8
	missing	6774	13.7	373	12.9	131	11.6	7278	13.6
Vital status	Alive	33,431	67.4	1911	66.1	688	60.8	36,030	67.2
	Dead	3526	7.1	189	6.5	63	5.6	3778	7.0
	Migrant	12,631	25.5	789	27.3	381	33.7	13,801	25.7

### Exposure assessment

**Fig. 9:**
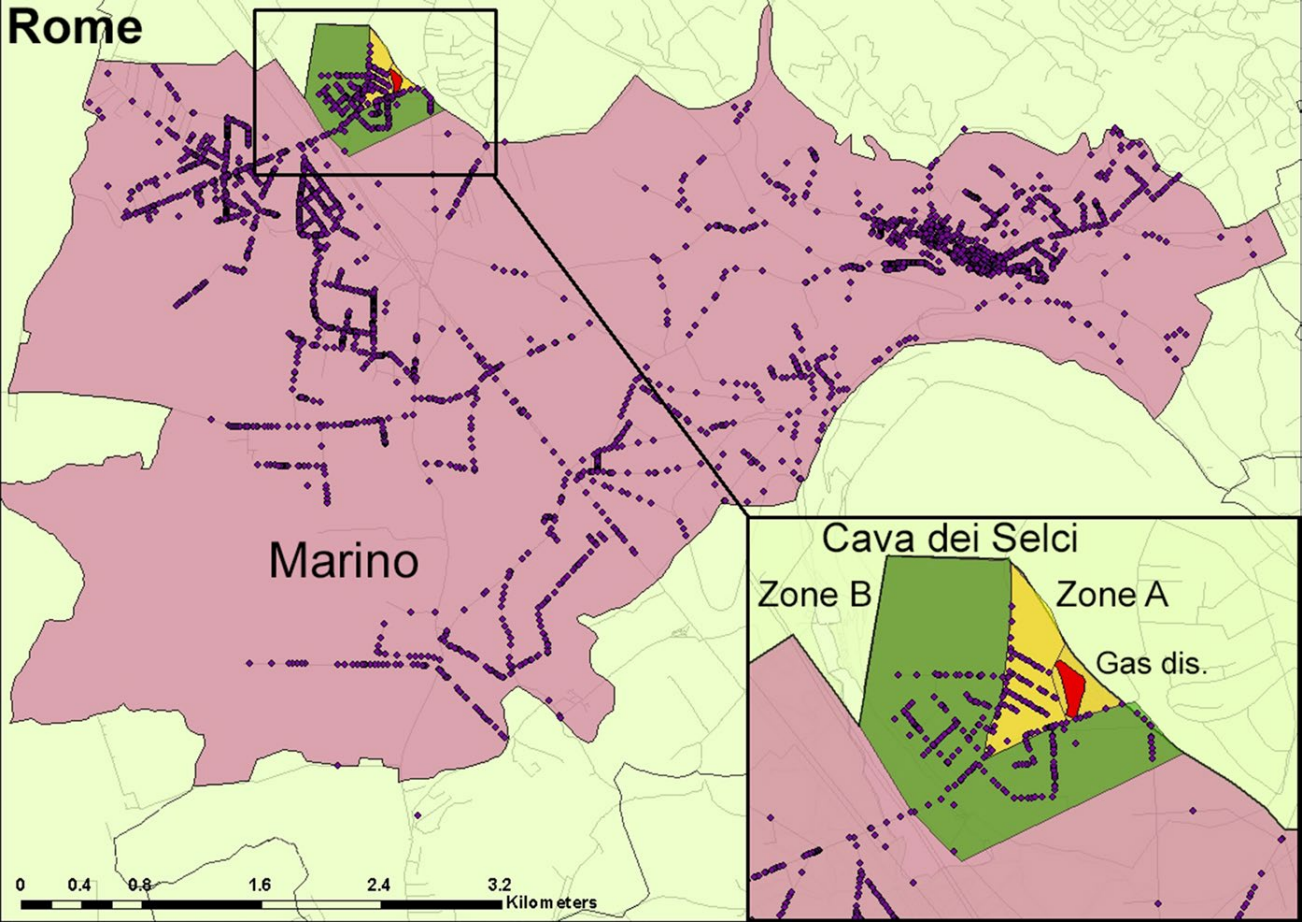
The three residential zones of the epidemiologic study: Cava dei Selci-zone A (yellow area); Cava dei Selci-zone B (green area); the reference zone of Marino municipality (pink area); red area is the gas discharge zone; dots = GIS geocoded addresses of residents

According to the emission levels of CO_2_ and H_2_S presented in the first part of the paper, the study area was divided in: zone A (69,105 m^2^) closer to the gas emission site, with the highest level of exposure; zone B (398,574 m^2^) surrounding zone A, with medium level of exposure and the reference zone of Marino municipality, with no emissions of CO_2_ and H_2_S (Fig. 9). An exposure level (high, medium, reference) was assigned to each subject in the cohort, based on the residential address; no individual estimation of the exposure was assigned. As made in the New Zealand—Rotorua study (Bates et al., 2002 and references therein), residents in each zone were considered as uniformly exposed to H_2_S and CO_2_.

### Outcomes definition and statistical analysis

Cause-specific mortality database (ReNCaM, 2010) and Healthcare Emergency Information System (HEIS, 2010) provided all information on mortality for the period 1996–2008 and on Emergency Room Visits (ERVs) for the period 2003–2008. Thanks to a record-linkage procedure between the cohort and the health databases, the cause of each death and the early symptom of each outpatient admission were assigned.

The mortality outcomes considered in the study are: all causes (ICD-9-CM 001-999), all cancers (ICD-9-CM 140-239), diseases of central nervous system (ICD-9-CM 330–349), cardiovascular disease (ICD-9-CM 390-459), ischaemic heart disease (ICD-9-CM 410-414), acute myocardial infarction (ICD-9-CM 410) and respiratory disease (ICD-9-CM 460-519). Mortality analysis was conducted by comparing the risk of residents in zone A, zone B and zone A + B with the reference zone, using a Cox model (HR, 95% CI) adjusted for age, socio-economic position and stratified by calendar period.

For the analysis of ERV, the outcomes considered are referred to the early symptoms at the moment of the outpatient admission: symptoms of nervous system, cardiovascular symptoms, dyspnoea, chest pain, pre-cordial pain, alteration of cardiac rhythm and intoxication. A repeated measure was used to compare the risk to have an access to ED for residents in zone A, zone B and zone A + B with the reference zone. For each participant, all the admissions to the ED during the period 2003–2008 were considered except those happened within seven days from the previous one. The Cox model follows the approach suggested by Prentice et al. (1981). The set of subjects at risk of undergoing the *k*th admission for a given case, in a generic instant *t*, is constituted by all the subjects under observation at time *t* who have previously had the (*k*-1)th hospitalization for the same cause. The model is adjusted for age and socio-economic position and stratified by calendar period and risk class. The risk class represents the order of admission for which the subject is at risk at any given time during the follow-up. At the beginning of the follow-up, everyone is at risk to undergo the first ERV; for this reason, they will have the same risk class, which is equal to 1; people who have had an ERV during the study period will be at risk to undergo the second admission, so their risk class will be equal to 2 and so on.

### Results of the epidemiological study

A total of 53,609 residents were geocoded and enrolled in the cohort in 1996 (51.2% women). Cava dei Selci-zone A, the closest to the gas emission, included 1132 residents (2.1%), Cava dei Selci-zone B included 2889 residents (5.4%), and the reference site of Marino municipality included all the other residents (Fig. 9).

In Table 4, the characteristics of the study cohort by residence zone, gender, vital status, age and socio-economic position are reported. The percentage of people older than 65 years decreases as getting closer to the gas discharge (7.3% in zone A, 9.7% in zone B and 10.5% in the reference zone). The socio-economic position differs significantly in the three zones: 83% of people living in zone A and 63% of people living in zone B have a middle-low or low socio-economic position, in contrast with the 25.6% of the reference group. In the whole Cava dei Selci (zone A + B), the high socio-economic class is absent, and in zone A also the middle-high class lacks. During the study period, 3778 people died and the crude percentage of deaths was lower in zone A than in the reference zone (5.6% and 7.1%, respectively). Since 1996, 13,801 people (25.6% of the cohort) moved from Marino to another municipality and only 36,030 people were identified as residents at the end of the follow-up in 2008.

The association between residence zone and mortality is shown in Table 5. Comparing mortality in zone A with that in the reference zone, an increased risk of mortality for all causes is found in men but not in women (HR 1.28, 95% CI 0.92–1.79 among men and HR 1.02, 95% CI 0.69–1.51 among women). In particular, an increased risk of mortality for diseases of central nervous system is found for men in zone A (HR 5.82, 95% CI 1.27–26.56) sustained also for the total of resident men in zone A + B (HR 2.93 95% CI 1.12–7.70). Indication of excesses of risks for cardiovascular diseases and myocardial infarction is found in men residents in zone A (HR 1.60, 95% CI 0.95–2.70 and HR 2.11, 95% CI 0.91–4.90, respectively). For women living in zone A + B, an increased risk of mortality for ischaemic heart diseases and acute myocardial infarction is found (HR 1.51, 95% CI 0.95–2.41 and HR 1.56, 95% CI 0.8–3.05, respectively). With respect to cancer mortality, no evidence is found in zone B, whereas a slight incidence of 1.24% (95% CI 0.61–2.52) for women and 1.11% (95% CI 0.62–1.99) for men is found in zone A (Table 5). However, cancer mortality is not representative of cancer incidence except for severe cases at later stages.

**Table 5:** Associations (hazard ratios, HR) between residence zone and causes of specific mortality in the cohort of Marino by gender (1996–2008)

Cause of death (ICD-9-CM)	Municipality of Marino									
	Reference zone	Zone B			Zone A			Zone A + B		
*Men*	no.	no.	HR	CI 95%	no.	HR	CI 95%	no.	HR	CI 95%
All causes (001–999)	1774	109	0.95	(0.78–1.16)	37	1.28	(0.92–1.79)	146	1.01	(0.85–1.21)
All cancers (140–239)	635	32	0.78	(0.54–1.11)	12	1.11	(0.62–1.99)	44	0.84	(0.62–1.15)
Central nervous system diseases (330–349)	24	4	2.37	(0.78–7.24)	2	5.82	(1.27–26.56)	6	2.93	(1.12–7.7)
Cardiovascular disease (390–459)	622	40	1.04	(0.75–1.44)	15	1.60	(0.95–2.7)	55	1.14	(0.86–1.52)
Ischaemic heart disease (410–414)	251	14	0.85	(0.49–1.47)	7	1.60	(0.74–3.45)	21	1.01	(0.64–1.59)
Acute myocardial Infarction (410)	158	10	0.98	(0.51–1.88)	6	2.11	(0.91–4.9)	16	1.22	(0.71–2.08)
Respiratory disease (460–519)	112	8	0.97	(0.46–2.01)	2	0.98	(0.24–4.05)	10	0.97	(0.5–1.89)
*Women*										
All causes (001–999)	1752	80	0.93	(0.74–1.16)	26	1.02	(0.69–1.51)	106	0.95	(0.78–1.16)
All cancers (140–239)	414	17	0.82	(0.5–1.34)	8	1.24	(0.61–2.52)	25	0.92	(0.61–1.39)
Central nervous system diseases (330–349)	35	1	0.52	(0.07–3.85)	0	0.00	–	1	0.39	(0.05–2.94)
Cardiovascular disease (390–459)	799	44	1.15	(0.85–1.57)	14	1.29	(0.76–2.21)	58	1.18	(0.9–1.56)
Ischaemic heart disease (410–414)	209	17	1.57	(0.94–2.6)	4	1.33	(0.49–3.63)	21	1.51	(0.95–2.41)
Acute myocardial Infarction (410)	98	8	1.62	(0.77–3.389)	2	1.34	(0.32–5.55)	10	1.56	(0.8–3.05)

The results of the ED analysis are shown in Table 6. The risk to have an emergency room visit for men is higher in zones A and B in comparison with the rest of Marino (HR 1.05, 95% CI 1.00–1.10 in zone B and HR 1.06, 95% CI 0.97–1.15 in zone A). A higher risk is found also for women resident in zone B (HR 1.07, 95% CI 1.02–1.13).

**Table 6:** Associations (hazard ratios, HR) between residence zone and ERVs in the cohort of Marino by gender (2003–2008)

Early symptoms	Municipality of Marino									
	Reference zone	Zone B			Zone A			Zone A + B		
*Men*	no.	no.	HR	CI 95%	no.	HR	CI 95%	no.	HR	CI 95%
Total	29,118	1886	1.05	(1.00–1.10)	644	1.06	(0.97–1.15)	2530	1.05	(1.00–1.10)
Symptoms of nervous system	254	26	1.47	(0.95–2.28)	8	1.57	(0.76–3.25)	34	1.50	(1.02–2.20)
Cardiovascular symptom	11,828	731	1.02	(0.95–1.11)	264	1.08	(0.95–1.23)	995	1.04	(0.97–1.11)
Dyspnoea	709	65	1.26	(0.95–1.69)	20	1.00	(0.57–1.76)	85	1.20	(0.92–1.57)
Chest pain	940	46	0.79	(0.59–1.07)	10	0.63	(0.35–1.16)	56	0.76	(0.58–1.00)
Pre-cordial pain	124	7	1.06	(0.51–2.22)	0	–	–	7	0.87	(0.41–1.83)
Alteration of cardiac rhythm	10,055	613	1.01	(0.93–1.10)	234	1.11	(0.97–1.28)	847	1.04	(0.96–1.11)
Intoxication	63	5	1.20	(0.47–3.05)	4	2.67	(0.94–7.57)	9	1.57	(0.78–3.15)
*Women*										
Total	27,030	1732	1.07	(1.02–1.13)	550	1.00	(0.92–1.09)	2282	1.04	(0.99–1.09)
Symptoms of nervous system	346	19	0.60	(0.23–1.17)	10	1.43	(1.58–7.57)	29	0.82	(0.52–1.31)
Cardiovascular symptom	12,321	879	1.17	(1.08–1.25)	247	0.99	(0.87–1.12)	1126	1.12	(1.05–1.2)
Dyspnoea	602	45	1.29	(0.93–1.8)	9	0.82	(0.44–1.53)	54	1.14	(0.84–1.54)
Chest pain	532	42	1.24	(0.88–1.73)	14	1.51	(0.88–2.58)	56	1.30	(0.97–1.75)
Pre-cordial pain	67	6	1.57	(0.65–3.76)	4	3.79	(1.27–11.3)	10	2.03	(0.99–4.17)
Alteration of cardiac rhythm	11,120	786	1.17	(1.09–1.26)	220	0.98	(0.86–1.12)	1006	1.12	(1.05–1.2)
Intoxication	69	4	0.68	(0.24–1.97)	1	0.46	(0.06–3.3)	5	0.62	(0.24–1.59)

In particular, the analysis shows a higher risk of ERV for symptoms of nervous system for men resident in zone A + B (HR 1.50, 95% CI 1.02–2.20). A risk indication is found for alteration of cardiac rhythm and intoxication for men living in zone A (HR 1.11, 95% CI 0.97–1.28 and HR 2.67, 95% CI 0.94–7.57, respectively). For women in zone A, main ERV risks are for symptoms of nervous system (HR 1.43, 95% CI 1.58–7.57) and pre-cordial pain (HR 3.79, 95% CI 1.27–11.30) was. Women resident in zone B and zone A + B have a higher risk to have an outpatient admission for cardiovascular symptoms (HR 1.17, 95% CI 1.08–1.25 and HR 1.12, 95% CI 1.05–1.20) and alteration of cardiac rhythm (HR 1.17, 95% CI 1.09–1.26 and HR 1.12, 95% CI 1.05–1.20) (Table 6).

## Discussion

All data coherently indicate that Cava dei Selci inhabited zone is severely exposed to gas hazard. Evidences are:

- The proximity to a natural gas discharge, with a permanent strong output of CO_2_ and H_2_S
- The high soil CO_2_ flux and the high air gas concentration of CO_2_ and H_2_S found both inside and outside several houses
- The illnesses caused by the gas (i.e. nausea, headache, stinging eyes, throat, etc.) complained by many residents, together with the unpleasant H_2_S rotten-eggs smell
- The presence of some houses and open spaces permanently interdicted to people by the Fire Brigade because of hazardous air gas concentrations
- The excess health risk for exposition to CO_2_ and H_2_S found in the cohort study of the resident population.

Besides the convective emission from fractures in the gas discharge zone, in the whole zone A gas is also diffusively released from the soil. Gas rising from depth infiltrates inside most houses, particularly in basements and ground floors. In the majority of zone A houses, gas concentration exceeds the acceptable long-term exposure range and, inside some of them, residents are exposed to concentrations above safety thresholds. The house-by-house indoor gas concentration survey (Table 1, Fig. 3) found that in 8% of the measurements CO_2_ exceeded 8.3 vol% with nearly 20% of values above 3 vol%. In 14% of the measurements H_2_S was above 6.4 ppm with 4% of the values exceeding 100 ppm. Similar and even higher indoor gas concentrations have been found in the houses evacuated in 2010 and 2018. The measured gas exposure level and the illnesses suffered by the residents are coherent with the health effects of different gas exposure described by IVHHN (2020) (see Tables S1 and S2 in Supplementary Materials).

An important result is the large time variation of gas concentration inside houses revealed by continuous monitoring (e.g. Figs. 5, 6). This implies that gas hazard can be assessed only by a long recording period, as discrete measurements could casually fall in low-degassing moments.

An increased risk of mortality for diseases of central nervous system has been found for men living in zones A and B exposed to H_2_S and CO_2_, compared to the reference zone. Moreover, an association between residence in exposed areas and risk of emergency room visit has been highlighted for several symptoms (symptoms of nervous system, cardiovascular symptoms, alteration of cardiac rhythm, pre-cordial pain and intoxication). According to the self-reported symptoms survey of Legator et al. (2001), low-level exposure of H_2_S is associated with an excess of respiratory symptoms, cardiovascular and central nervous system diseases. In New Zealand, it has been demonstrated that exposure to H_2_S can cause higher respiratory mortality risk, and higher morbidity risk for diseases of the peripheral nervous system (Bates et al., 1997, 1998 and 2002). Our analysis shows excesses of mortality and ERVs for nervous system diseases, but no evidence of any excess for respiratory diseases. Furthermore, several studies found elevated cancer risk among women living in zones exposed to gas emission (Hansell & Oppenheimer, 2004; Bates et al., 1997, 1998 and 2002). Kristbjornsdottir et al. (2012) analysed cancer incidence in a cohort of residents near an Iceland geothermal source and found that HR in the exposed area for all cancers was 1.22 (95% CI 1.05–1.42) as compared to the reference area. We could not consider cancer incidence as an outcome in our study since a cancer registry is not available in this area. Our results are limited to cancer mortality and in zone A the related HR for all cancers is higher for women (Table 5).

Age and socio-economic position were considered as confounders in the analysis. The socio-economic position is an area-based index and is assigned on a census-block level, and then the individual socio-economic position is undefined (Cesaroni et al., 2006). Similarly to other studies, other information on lifestyle of the study population is unknown. In our case, it would have been impossible to collect, from municipality data, other individual confounders.

A strength of our study consists in using a repeated measure Cox model to analyse the ER admissions of the cohort. This approach allowed considering several events for each subject under study avoiding the limitation of taking into account only one failure for subject. So we considered all the ER admissions occurred during the follow-up period. Since failure times of repeated events are correlated within subject (violating the independence assumption required in traditional survival analysis; Cleves, 1999), we assigned different baseline risks (risk class) according to the different recurrence of the events. Each risk category represents the risk of being admitted at any given time during the follow-up. The repeated measure model considers a greater number of events obtaining more statistical power, and allows to study chronic conditions and disease severity, usually related to repeated admissions to ER during the follow-up period.

Geochemical data and epidemiological results indicate a severe health gas hazard of the urbanized zone of Cava dei Selci and only by chance some residents suffered only illnesses before being evacuated, as the gas might have killed them.

## Conclusions

Cava dei Selci is the most important natural gas discharge of central Italy located in an urbanized context, near Rome. Anomalous values of diffuse soil CO_2_ flux have been found in the urbanized zone A and the area surrounding it, where a total soil CO_2_ flux of nearly 16 tonnes * day^−1^ has been estimated from 68,380 m^2^. Gas rising from depth infiltrates inside houses, mostly along connections with the underlying soil, such as electric sockets and hydraulic dumping. Drain grates and air bends connecting the crawl spaces underneath houses are sites of frequent outside gas emissions. High air concentrations of CO_2_ and H_2_S have been measured inside many residences, particularly in basements, four of which had to be evacuated for safety reasons. The epidemiological study found an increased risk of mortality for diseases affecting the central nervous system. The emergency room visits were related to symptoms of nervous system, cardiovascular symptoms, alteration of cardiac rhythm, pre-cordial pain and intoxication. Results are consistent with high exposures to H_2_S and CO_2_ and confirm that Cava dei Selci residents are affected by gas-related health problems.

About ten years ago, it seemed that regional and local authorities wanted to address seriously this problem, which was threatening nearly 4000 people, in particular the 1126 residents of the zone nearest to the natural gas discharge. The Civil Protection Department of Lazio Region convened a technical panel involving Marino municipality, national and regional health institutions, urban planning office, Fire Brigade and our scientific group (INGV and Epidemiological Department of Lazio). The problem was discussed in many meetings, and all possible solutions were analysed. It was clear that in almost the entire Cava dei Selci zone, the CO_2_ and H_2_S concentration inside residences was exceeding the admitted values for human health. Hazardous air gas concentration was frequently found also outdoor, and there was a health impact on local population. In other words, this zone should never have been urbanized because of adverse environmental conditions. A revision of the building regulatory plan of Cava dei Selci to prevent any new edification was considered by Marino municipality, but it was not actuated so far.

All possible remediation techniques were considered, including the installation of automatic monitoring systems with an alert system for air gas concentration inside residences and forced air ventilation systems. However, no action was undertaken to mitigate the risk, the most recurrent motivation being the estimated high cost and maintenance difficulty. Only an information campaign with a public meeting and distribution of a brochure with information on the gas hazard of the zone was organized in September 2010 (Carapezza et al., 2010c) and never repeated.

On 7 August 2010, the Lazio region issued an Emergency Declaration of natural calamity for Cava dei Selci because of hazardous gas emission and requested the technical and financial support of the National Civil Protection Department to reduce the risk, but unfortunately this support was not granted.

As a whole, data indicate that Cava dei Selci is one of the urbanized zones most exposed to endogenous gas hazard in the world. Geochemical monitoring continued up to 2012 and resumed in 2019 for volcanic surveillance purpose, whereas there is still a need of an epidemiological surveillance of the population. This study shows that natural gas emission sources can be as dangerous as the industrial ones, but they are more insidious because they are poorly known and underestimated.

## Supplementary Information

Below is the link to the electronic supplementary material.

Supplementary file1 (DOCX 13817 KB)

## Data Availability

Geochemical data are freely available in Earth-Prints INGV repository.

## Code availability

Geochemistry, http://hdl.handle.net/2122/13953
