# Supplementary material for: Health impact of natural gas emission at Cava dei Selci residential zone (metropolitan city of Rome, Italy)
Source: Environ Geochem Health. 2022 Mar 12;45(3):707–29. doi: 10.1007/s10653-022-01244-6 (PMC10014802; doi:10.1007/s10653-022-01244-6)
Supplement: Supplementary file 1 — Supplementary file1 (DOCX 13817 KB) [file 10653_2022_1244_MOESM1_ESM.docx]

**Supplementary Materials**

**Figures**

**
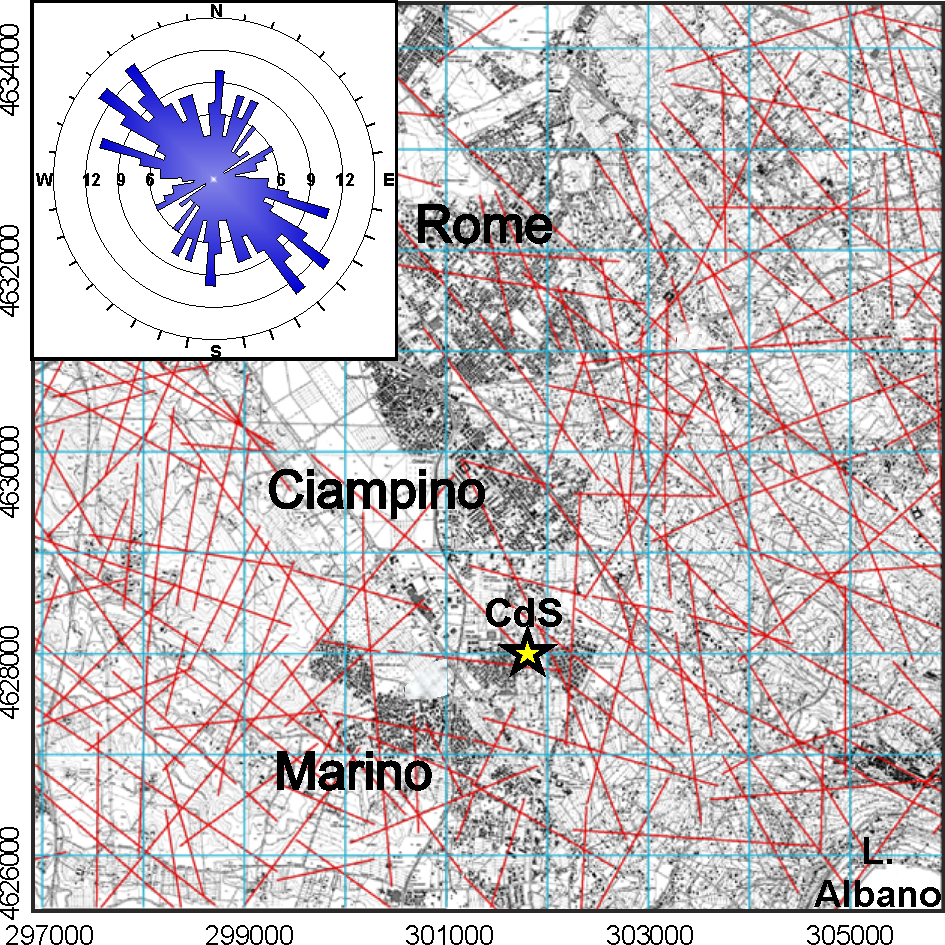
**

**Fig. S1** Morpho-structural map of the north-western zone of Colli Albani volcano, including Cava dei Selci (CdS, yellow star); the insert shows the related Rose diagram of the structural lineaments

**Fig. S2** Swelling and cracking caused by gas emission in a Via Maciocco house, March 2018. a) Caved loose material with sulphur incrustations under the swelling of the living room floor. b) Detached tiles outside the house entrance. c) Swelling and fractures in the concrete floor of the confining courtyard

**Tables**

| **Table S1.** Health effects of respiratory exposure to hydrogen sulphide (H_2_S) | | |
| --- | --- | --- |
| Exposure limits (ppm) | Health Effects |  |
| 0.008-0.2 | Olfactory threshold -“rotten eggs” smell detectable |  |
| 20 | Sense of smell to gas lost.  Concentrations tolerated for some hours without harm. |  |
| 20-50 | Eye irritation. |  |
| 50 | Prolonged exposure may cause pharyngitis and bronchitis. |  |
| 60 | Prolonged exposure may cause conjunctivitis and eye pain. |  |
| +150 | Irritation of upper respiratory tract.  Sense of smell lost. |  |
| 250 | Pulmonary oedema with risk of death. |  |
| 500 | Very dangerous, evacuation should occur well below this level. |  |
| 1000 | Loss of consciousness occurs. |  |
| 1000-2000 | Acute intoxication: symptoms include rapid breathing, distress, nausea, vomiting. May be rapidly followed by loss of consciousness, coma and cessation of breathing. |  |
| >2000 | Immediate loss of consciousness and high probability of death. |  |
| After IVHHN (2020) and references therein. | | |
| **Table S2.** Health effects of respiratory exposure to carbon dioxide (CO_2_) | | |
| Exposure limits (% in air) | Health Effects |  |
| 2-3 | Unnoticed at rest, but on exertion there may be marked shortness of breath. |  |
| 3 | Breathing becomes noticeably deeper and more frequent at rest. |  |
| 3-5 | Breathing rhythm accelerates. Repeated exposure provokes headaches. |  |
| 5 | Breathing becomes extremely laboured, headaches, sweating and bounding pulse. |  |
| 7.5 | Rapid breathing, increased heart rate, headaches, sweating, dizziness, shortness of breath, muscular weakness, loss of mental abilities, drowsiness, and ringing in the ears. |  |
| 8-15 | Headache, vertigo, vomiting, loss of consciousness and possibly death if the patient is not immediately given oxygen. |  |
| 10 | Respiratory distress develops rapidly with loss of consciousness in 10-15’. |  |
| 15 | Lethal concentration, exposure to levels above this is intolerable. |  |
| >25 | Convulsions occur and rapid loss of consciousness ensues after a few breaths. Death will occur if level is maintained. |  |
| After IVHHN (2020) and references therein. | | |
